# The Future of TDR: It Will Share the R&D Landscape with Other National and Multilateral Agencies

**DOI:** 10.1371/journal.pntd.0000309

**Published:** 2008-11-25

**Authors:** Anthony Mbewu

**Affiliations:** Medical Research Council of South Africa, Tygerberg, South Africa; *PLoS Neglected Tropical Diseases*, United States of America

The Fourth External Review of the Special Programme for Research and Training in Tropical Diseases (TDR) took place between February 2005 and May 2006, led by Professor Abdallah Daar and colleagues [1]. Since its establishment in 1978, TDR has drawn heavily on the recommendations of its external reviews in order to shape its strategy and structures going forward [2]. This fourth review was particularly pertinent given the dramatically changed global health landscape that TDR finds itself in at the beginning of the 21st century.

Having experienced great success in research capacity strengthening and product development for neglected diseases over the years, TDR now shares the space with many other national and multilateral agencies in these two fields. At the time of the Fourth External Review, it needed to carefully examine, consult on, and reshape its strategy in order to work effectively in this new milieu.

The two articles in this issue of *PLoS Neglected Tropical Diseases* summarise the Fourth External Review [1] and map out the response of TDR in its new Ten Year Strategy and Business Plan [3]. The plan was approved by TDR's Joint Coordinating Board (JCB) in June 2007, and the strategy is now in the first stages of implementation, which began in January 2008.

The Fourth External Review mapped out the strengths and weaknesses of TDR. From my point of view as the President and Chief Executive Officer of a Medical Research Council (MRC) in a disease-endemic country (South Africa), the review's assessment of TDR's strengths and weaknesses seemed valid and insightful. Daar and his colleagues then recommended that TDR undertake a major re-orientation and stakeholder engagement exercise, and reconfigure its work into four functional areas: stewardship, research advocacy, and coordination; research and development for physical products; expanded intervention research; and research capacity strengthening for the future.

TDR has largely followed these recommendations under the banner of its new vision—“to foster an effective global research effort on infectious diseases of poverty in which disease-endemic countries play a pivotal role” [2].

The new vision embodies the review recommendation of an increased emphasis on “needy populations” as compared to “neglected diseases”. The vision also includes a move towards more transdisciplinary work, including addressing the social as well as biomedical determinants of health.

The strategy illustrates this new approach through a diagram of the research continuum that stretches from basic research through product development to intervention and implementation research and ultimately health impact [3]. Within this continuum, TDR will strategically focus on knowledge management (linked to its stewardship function); capacity building (linked to its empowerment function); and neglected areas in research.

The aim of this focus is to ensure that TDR finds a role for itself filling gaps in the new global landscape, and forging strong partnerships with other global players as well as with research institutions in disease-endemic countries. Thus in South Africa, TDR has forged strong collaborations in the past few years on drug availability studies for tuberculosis drugs, as well as phase III clinical trials of treatment shortening regimens for tuberculosis infection. The strategy also shows how TDR's new functions map out against the research continuum, with expert scientific advisory committees convened to support the three functions of stewardship, empowerment, and research “business line activities”.

TDR is implementing the review recommendation that “all stakeholders [support] TDR to evolve and grow to a renewed mandate” [1] by becoming more responsive to stakeholder issues, including changes in the processes of the JCB in order to respond to wider constituencies than are represented on the JCB. The review criticism that TDR was “over-administrated and under-managed” [1] is being addressed in part through decentralising managerial and administrative authority, and also through initiatives at the World Health Organization (the legal executing agency for TDR) to streamline its excessive bureaucracy. However, TDR's governing bodies chose not to decentralise its activities to regional centres. TDR and the co-sponsoring agencies will actively support the development of national research policies.

In the past ten years, TDR has established many product development activities, but will in future focus on research and development for physical products for very neglected diseases that others are not addressing adequately. TDR will remain involved in basic hypothesis-driven research, but will increase its emphasis on expanded intervention research where there are many diverse actors. Once drugs and diagnostics are developed, research is needed to examine their impact on individual and population health, which in turn is dependent on “micro” and “macro” factors. The micro factors include the behavioural determinants of medicine use and adherence. The macro factors include the health systems that are necessary to deliver these medicines, including the health economics of sustainable supply systems. Operational research is needed to evaluate and improve the implementation of new health programmes.

In conclusion then, from the perspective of the manager of a national research institution in a disease-endemic country, TDR seems poised and able to realize the vision quoted above. It has already established collaborations with my research institution, the MRC, that involve equal partnerships with MRC scientists playing a critical role in both phase II and III clinical trials. We look forward to long and expanded collaborations between TDR and all disease-endemic countries in understanding the biomedical and social pathogenesis of infectious disease, and developing new tools for diagnosis and treatment of those diseases.
